# New species and record of *Dodecaceria* (Annelida: Cirratulidae) the Biological Reserve of Rocas Atoll, Brazil, the only atoll in the South Atlantic Ocean

**DOI:** 10.1371/journal.pone.0293087

**Published:** 2023-10-23

**Authors:** Christine Ruta, Davi Moreira Mundim, Roberta Freitas, Rannyele P. Ribeiro

**Affiliations:** 1 Departamento de Zoologia, Instituto de Biologia, Centro de Ciências da Saúde, Universidade Federal do Rio de Janeiro, Rio de Janeiro, Rio de Janeiro, Brazil; 2 Programa de Pós-Graduação em Zoologia, Museu Nacional, Universidade Federal do Rio de Janeiro, Rio de Janeiro, Rio de Janeiro, Brazil; 3 Department of Biology, Washington University in St. Louis. St. Louis, Missouri, United States of America; UMinho CBMA: Universidade do Minho Centro de Biologia Molecular e Ambiental, PORTUGAL

## Abstract

The polychaete Family Cirratulidae is one of the most abundant and diverse groups of Annelida, although it remains poorly known worldwide. *Dodecaceria* Ørsted, 1843 is one of the least described genera of Cirratulidae. The present report is the first taxonomic study of the genus *Dodecaceria* for the Brazilian coast. Cirratulidae were collected at Rocas Atoll, the first Brazilian marine protected area and the only atoll in the South Atlantic Ocean. We described one new species, *Dodecaceria zelinhae* n. sp., and a new record of *D*. *dibranchiata* Blake & Dean, 2019, previously only known from Panama. The new species is distinguished from other *Dodecaceria* species by having lateral tentacles, a smooth peristomium, 3–5 pairs of branchiae, hooks from chaetiger 11 in notopodia and 9 in neuropodia. *Dodecaceria dibranchiata*, a Caribbean species, is here recorded for the first time in the South Atlantic Ocean.

## Introduction

The polychaete Family Cirratulidae Ryckholt, 1851, is one of the most abundant and diverse groups of the phylum Annelida. Regarding genera composition within the Cirratulidae family, two perspectives differ in their number. Taxonomic studies indicate the existence of 11 genera [1], while recent phylogenetic studies include Ctenodrilinae Kennel, 1882 as a subfamily within Cirratulidae, totaling 16 genera [2]. Cirratulids occur in all oceans and depths, on surfaces or sub-surfaces of soft bottoms, although some species of the genus *Dodecaceria* Ørsted, 1843 are found on hard bottoms [3]. Initial inclusion of Cirratulidae in a sequence database (18S rRNA) occurred with *Dodecaceria concharum*, as documented by Winnepenninckx *et al*. in 1998 [4]. This specific sequence was subsequently used to gain insights into the relationships among larger taxonomic groups within the phylum Annelida, playing a crucial role in the study of Annelida phylogeny [5, 6].

Currently, *Dodecaceria* comprises approximately 20 valid species [1]. The genus is a hard bottom cirratulid and is readily characterized, among other features, by having a single pair of dorsolateral tentacles, branchial filaments generally restricted to anterior chaetigers, and acicular hooks simple, spoon shaped, or sometimes serrated [7].

Like other cirratulids, the genus is confusing due to the superficial morphological similarity of species. Thus, for example, cirratulids lose important taxonomic characters during collection [3, 8], such as in *Dodecaceria*, when frequently removed from their tubes, specimen handling can result in the fragmentation of the body at either the posterior or anterior end [9]. Other factors that can contribute to taxonomic misidentification are reproductive characteristics. *Dodecaceria* species have a complex life cycle characterized by sexual and asexual reproduction [1], which often makes it difficult to correctly distinguish different states of development or stage of regeneration.

Studies of *Dodecaceria* are rare, and the majority of species described are found in the North Atlantic Ocean [1]. Although there has been increasing work on the Cirratulidae in the South Atlantic Ocean [10–14], including Brazilian waters [12, 13]. However, *Dodecaceria* is poorly known in this region. Only three species were reported to the South Atlantic Ocean: *Dodecaceria meridiana* Elías & Rivero, 2009 in the littoral of Argentina [14], *D*. *capensis* Day, 1961 was reported in a checklist by Praia do Seixas, Paraiba State [15], and *Dodecaceria concharum* Ørsted, 1843 in a master’s thesis for the São Paulo coast [16]. To date, no taxonomic work has been published for *Dodecaceria* in Brazil.

The present work reports *Dodecaceria* from Rocas Atoll, the only atoll in the South Atlantic Ocean and one of the world’s smallest atolls [17]. Rocas Atoll was designated a Brazilian Marine Conservation Unity in 1979 [18]. The Biological Reserve of Rocas Atoll is a protected area where scientific research is the only permitted human activity [19]. Herein, we describe a new species, *Dodecaceria zelinhae* n. sp., and a new record *Dodecaceria dibranchiata* Blake & Dean, 2019 from the Rocas Atoll.

## Material and methods

**Fig 1:**
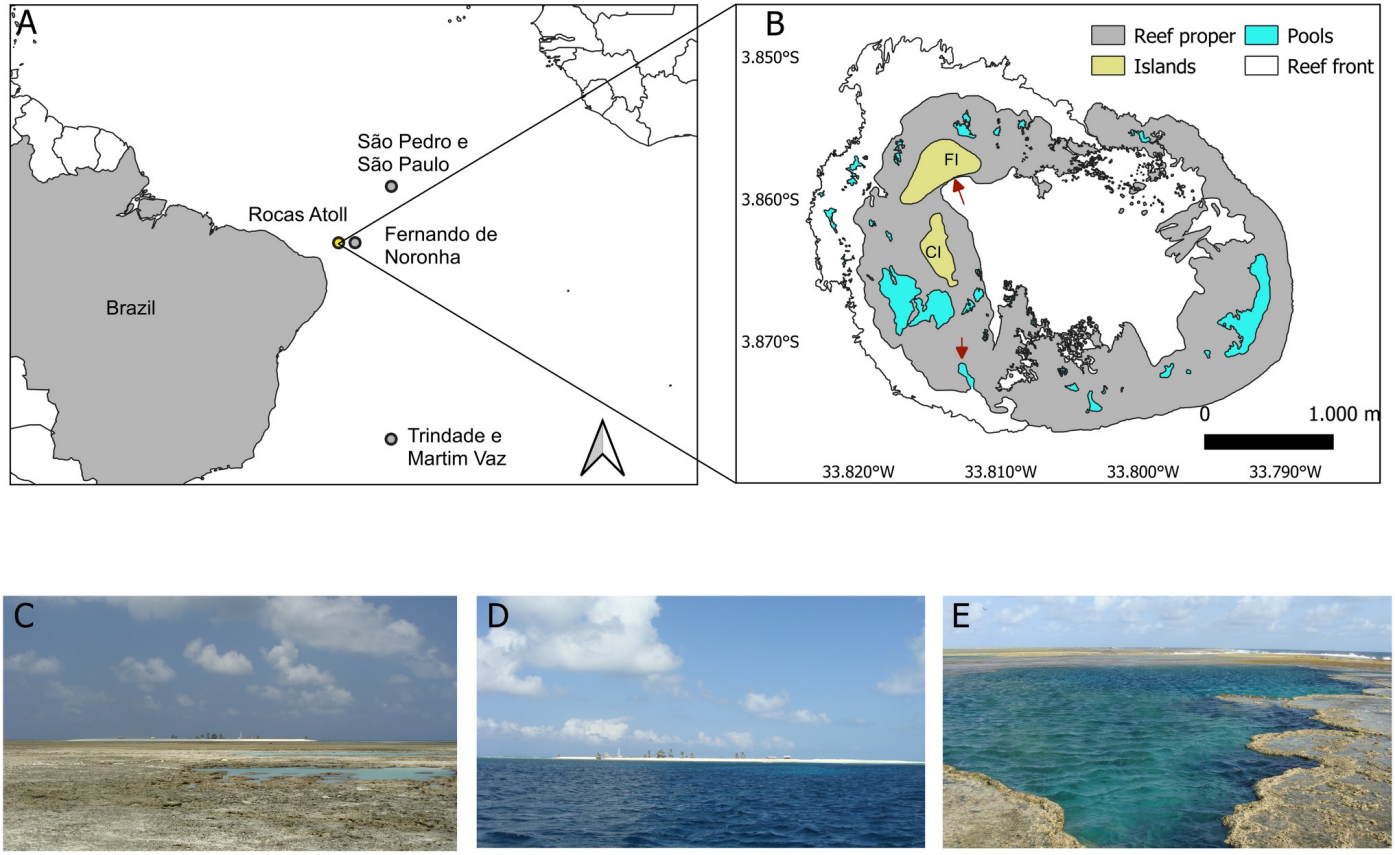
Map of the study area, Rocas Atoll, Brazil. (A) Brazilian oceanic islands location; (B) Rocas Atoll map; (C) Atoll view during low tides; (D) Atoll view during high tides; (E) Tidal poll.

The Biological Reserve of Rocas Atoll is located 266 km northeast of the coastal city of Natal, in the State of Rio Grande do Norte, and 148 km from the Fernando de Noronha Archipelago, in the State of Pernambuco (Fig 1A). The atoll has a volcanic origin and coralline formation, its largest axis is approximately 3.7 km long, and the shortest axis is 2.5 km long. There are two islands, Farol and Cemitério, encompassing 7.2 km^2^ of emerging area inside the atoll ring [20]. Except for the two islands, the atoll area is submerged during high tides, and several pools are exposed during low tides (Fig 1B). Tide pools vary in depth from 0.5 m to more than 7 m [20].

The specimens of *Dodecaceria* were collected between 13 September and 5 October 2015. Samples of hard bottom were taken in areas mainly composed of coralline rocks in two different environments: hard bottoms near Farol Island (0–2 m depth) and a tidal pool named Piscina Podes Crer (0–7 m depth). Specimens were removed after manual fragmentation of hard bottom, and after fixed in 4% formalin in seawater and preserved in 70% ethanol. For a detailed observation of the characters, the specimens were examined and measured using the Zeiss Stemi SV 11 stereomicroscope and Zeiss Axio Lab A1 microscopes. In addition, selected specimens underwent scanning electron microscopy (SEM) at the Laboratório de Imagem e Microscopia Óptica e Eletrônica, Instituto de Biologia, Universidade Federal do Rio de Janeiro (UFRJ), using a HITACHI TM3030 plus scanning electron microscope. Specimens for SEM were dehydrated in a graded series of increasing concentrations of ethanol (92%), critical point-dried, and coated with ~35 nm of gold. The holotype and paratypes of the newly described species were deposited at the Annelida Collection of the Museu Nacional do Rio de Janeiro (MNRJP), UFRJ, Rio de Janeiro, Brazil.

The following abbreviations are used here: br, branchiae; Cons, constriction; neP, neuropodium; noP, notopodium; per, peristomium; pr, prostomium; pyg, pygidium; Reg, regeneration; and tn, tentacle.

## Nomenclatural acts

The electronic edition of this article conforms to the requirements of the amended International Code of Zoological Nomenclature. Hence, the new names contained herein are available under that Code from the electronic edition of this article. This published work and the nomenclatural acts are registered in ZooBank. The ZooBank LSIDs (Life Science Identifiers) can be resolved, and the associated information viewed through any standard web browser by appending the LSID to the prefix ″http://zoobank.org/″. The LSID for this publication is urn:lsid:zoobank.org:pub: EEBDA733-911F-4499-AF9D-52AED22A35CB. The electronic edition of this work was published in a journal with an ISSN, has been archived, and is available from the following digital repositories: PubMed Central, LOCKSS.

## Results

### Systematics

#### Family Cirratulidae Ryckholt, 1851

Diagnosis. Body elongated, cylindrical. Thoracic region often expanded, abdominal region narrow or moniliform, sometimes expanded in the pre-pygidial region. Prostomium narrow and conical or wide, without appendages, eyespot present or absent, and dorsolateral paired nuchal organs. Peristomium smooth or with annular rings. Grooved tentacles present as a single pair or multiple from the peristomium or subsequent anterior chaetigers. Branchiae long, starting from the peristomium or first chaetigers, inserted dorsally and usually present up to the abdominal region. Parapodia birramous with rudimentary lobes. Chaetae simple, including capillaries, acicular spines, and bidentate or multidentate hooks. Pygidium with a simple lobe, sometimes with sub-anal disk or terminal cirrus. Pharynx ventral, unarmed [1].

#### Genus *Dodecaceria* Ørsted, 1843

*Type species*. *Dodecaceria concharum* Ørsted, 1843 [7], by monotypy

*Type locality*. North Atlantic Ocean, Denmark [7].

*Diagnosis*. Prostomium conical or rounded on anterior margin, forming a hood over the mouth. Peristomium short or long, with or without annular rings. A pair of grooved tentacles arising laterally or dorsolaterally in the peristomium. One to several pairs of branchial filaments extending over one to few anterior segments. Chaetae simple, including capillaries and stout, and acicular hooks simple, chisel or spoon-shaped, or sometimes serrated [1].

*Remarks*. The taxonomy of the genus *Dodecaceria* poses difficulties, partly due to the loss of the type specimen and the limited description of the type species. Ørsted [7] provides a brief overview of the type species, *Dodecaceria concharum*, discovered in Danish waters, although specific details about the location and date of collection of the type specimens were n not specified. These challenges are further compounded by the confusion surrounding the taxonomic identification of *Dodecaceria* species. Not only is it challenging to determine morphological characters, but there has also been a historical lack of taxonomic information available for both the type species and subsequent species within the genus. Subsequently, Caullery and Mesnil [21] reported the presence of *D*. *concharum* in the commune La Hague, Manche, France, and the morphological examination of the specimens revealed the existence of three distinct morphotypes. Two of these morphotypes were later recognized as specimens of *D*. *ater* (Quatrefages, 1866) [22, 23]. Adding to the complexities of *Dodecaceria* taxonomy, many species of *Dodecaceria* exhibit different modes of asexual reproduction. This involves their remarkable ability to regenerate their entire bodies [22, 23]. Asexual reproduction in *Dodecaceria* occurs through intriguing mechanisms, such as regenerating body parts after fissioning. This process is also observed in other annelids, such as syllids [24, 25]. Examples of fission have been reported in several *Dodecaceria* species, including *D*. *berkeleyi* Knox, 1971 [26, 27], *D*. *diceria* Hartman, 1951 [28], *D*. *fimbriata* [29, 30], *D*. *meridiana* [31], *D*. *pulchra* [23, 32], *D*. *sextentaculata* [23], and *D*. *concharum* [21, 22]. In the case of *D*. *concharum*, the larger segments of the trunk break off individually and regenerate into new individuals [23]. Due to the morphological differences that can occur during asexual reproduction or regeneration, there have been historical records of several misidentifications among *Dodecaceria* species. Consequently, Gibson [27] recommended that the taxonomy of the group should consider reproductive modes. Currently, the genus *Dodecaceria* comprises approximately 20 valid species [1], of which five are known to occur in South Atlantic waters. In the present work, a new occurrence of *D*. *dibranchiata* and a new species, *D*. *zelinhae* n. sp., are recorded for Rocas Atoll.

#### *Dodecaceria dibranchiata* Blake & Dean, 2019

(Fig 2).

**Fig 2:**
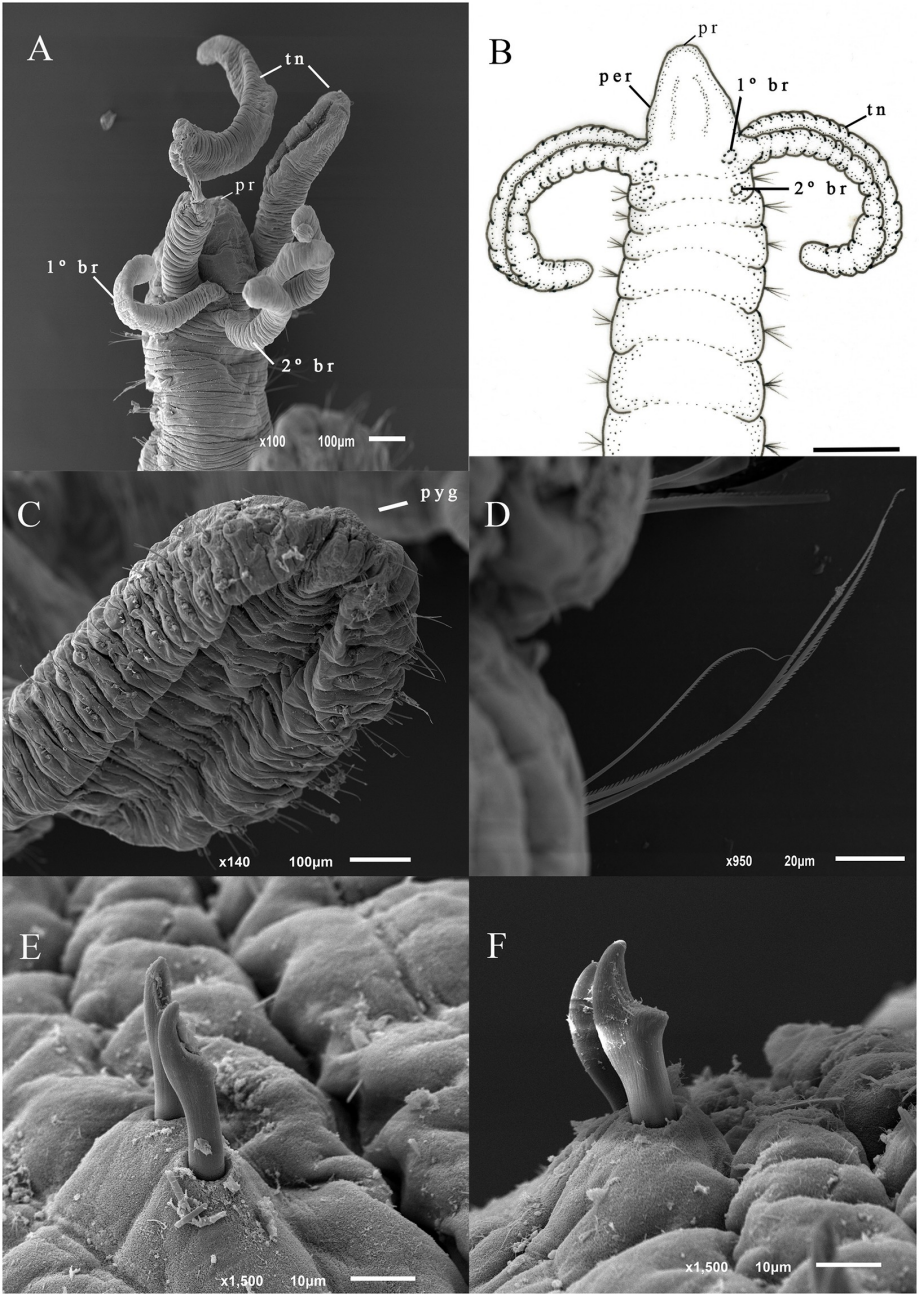
*Dodecaceria dibranchiata*. (A) Anterior end, dorso-lateral view; (B) Anterior end, dorsal view; (C) Posterior end, ventro-lateral view; (D) Notopodial serrated edge capillaries from anterior region; (E) Neuropodial hooks from anterior region; (F) Notopodial hooks from anterior region. Scale bars: 400 μm (B).

*Type locality*. Caribbean Sea, Panama, Golfo de San Blas, Playita Point [9].

*Material examined*. BRAZIL: Rio Grande do Norte, Rocas Atoll, Farol Island–-3.860350°S, -33.815775°W, intertidal, 09/21/15, five inds., three complete (MNRJP-007565) and two incomplete (MNRJP-007566); 09/10/15, two inds., prepared for SEM.

*Description*. Largest specimen with 70 chaetigers, 15.8 mm long (from prostomium to pygidium). Anterior chaetigers wider than long (Fig 2A and 2B), and posterior segments are shorter and flattened dorsoventrally (Fig 2C). Color in alcohol pales with brown pigments in the posterior and anterior regions. Prostomium conic, peristomium expanded (Fig 2A and 2B). Eyespots absent, nuchal organs not observed. Tentacles inserted laterally in the peristomium. Two pairs of branchiae, first pair between the peristomium and the chaetiger one and the second pair on the first chaetiger (Fig 2B). Capillary setae with serrated edges are present throughout the entire body (Fig 2D). Neuropodium with 1–3 capillaries up to the 11th chaetiger, capillaries absent from chaetiger 12, up to the last two neuropodial fascicles with one capillary. Notopodium with 2–3 capillaries up to the 12th chaetiger, 1–3 capillaries from 13th chaetiger, the last notopodial fascicles without capillaries. Neuropodial hooks from 10th chaetiger up to the last chaetiger, 1–2 hooks per fascicle. Neuropodial hooks shorter, wider, with deep apical concavity (Fig 2E). Notopodial hooks from the 13th to the 68th chaetiger, 1–3 hooks per fascicle. Notopodial hooks long, slender, with shallow apical concavity, and somewhat spoon-shaped (Fig 2F). Pygidium conic (Fig 2C).

*Habitat*. Coral reefs, 0–4 m depth [9]. In the present study, hard bottom and coral reefs, intertidal depths.

*Distribution*. Caribbean Sea (Panama) [9]; Rocas Atoll (Brazil), in the present study.

*Remarks*. The specimens from Rocas Atoll have the same diagnostic characters as *Dodecaceria dibranchiata* Blake & Dean, 2019, including only two pairs of branchiae, from the peristomium and the first chaetiger, respectively. Additionally, the peristomium is smooth and elongate, and all hooks are spoon-shaped. *D*. *dibranchiata* was originally described from the Caribbean Sea (Panama) in coral reefs. The species *Dodecaceria diceria* Hartman, 1951 and *Dodecaceria laddi* Hartman, 1954 are also characterized by two pairs of branchiae. *Dodecaceria diceria* differs from *D*. *dibranchiata* from Rocas Atoll and *D*. *dibranchiata* by having hooks with small grooves in the geniculate region [33] *Dodecaceria laddi* differs from *D*. *dibranchiata* by having a transversal groove between the prostomium and the peristomium and an annular ring in the peristomium [34]. Furthermore, the species differ in terms of habitat and type locality, *D*. *diceria* was described in association with gastropod shells off the coast of Florida collected 214 m deep [35]. In comparison, *D*. *dibranchiata* from Rocas Atoll was collected on hard bottom and coral reefs in intertidal depths, and *D*. *dibranchiata* Blake & Dean, 2019 was described in association with corals off the coast of Panama 0–4 m deep [9]. *D*. *laddi* was described from the Marshall Islands in the Pacific Ocean [34]. In contrast, *D*. *dibranchiata* from Rocas Atoll, and *D*. *dibranchiata* Blake & Dean, 2019 were described and collected in the Atlantic Ocean. Morphological variation regarding the number of branchia pairs remains unclear for *D*. *diceria* due to the lack of information on the number of branchiae and examined specimens in the original description [33]. A specimen (MNRJP-007565) exhibited a possible sign of regeneration, with a narrowing observed in chaetiger 19. Another evidence of regeneration was observed in *D*. *dibranchiata* from Rocas Atoll, as indicated by lighter pigmentation in the posterior and anterior ends in one specimen. The ability to perform anterior and posterior regeneration strongly suggests that this species can reproduce by paratomic fission. The process of asexual reproduction involving regeneration is already recognized in different other species of *Dodecaceria*, such as *D*. *berkeleyi* [26, 27], *D*. *concharum* [21–23], *D*. *meridiana* [31], *D*. *pulchra* [23, 32]. This is the first record of regeneration and asexual reproduction for *Dodecaceria dibranchiata*.

#### *Dodecaceria zelinhae* n. sp. Mundim, Freitas & Ruta

**urn:lsid:zoobank.org**:**act:3D8BC5B6-BE22-42FD-B99D-6D828592390B**

(Fig 3).

**Fig 3:**
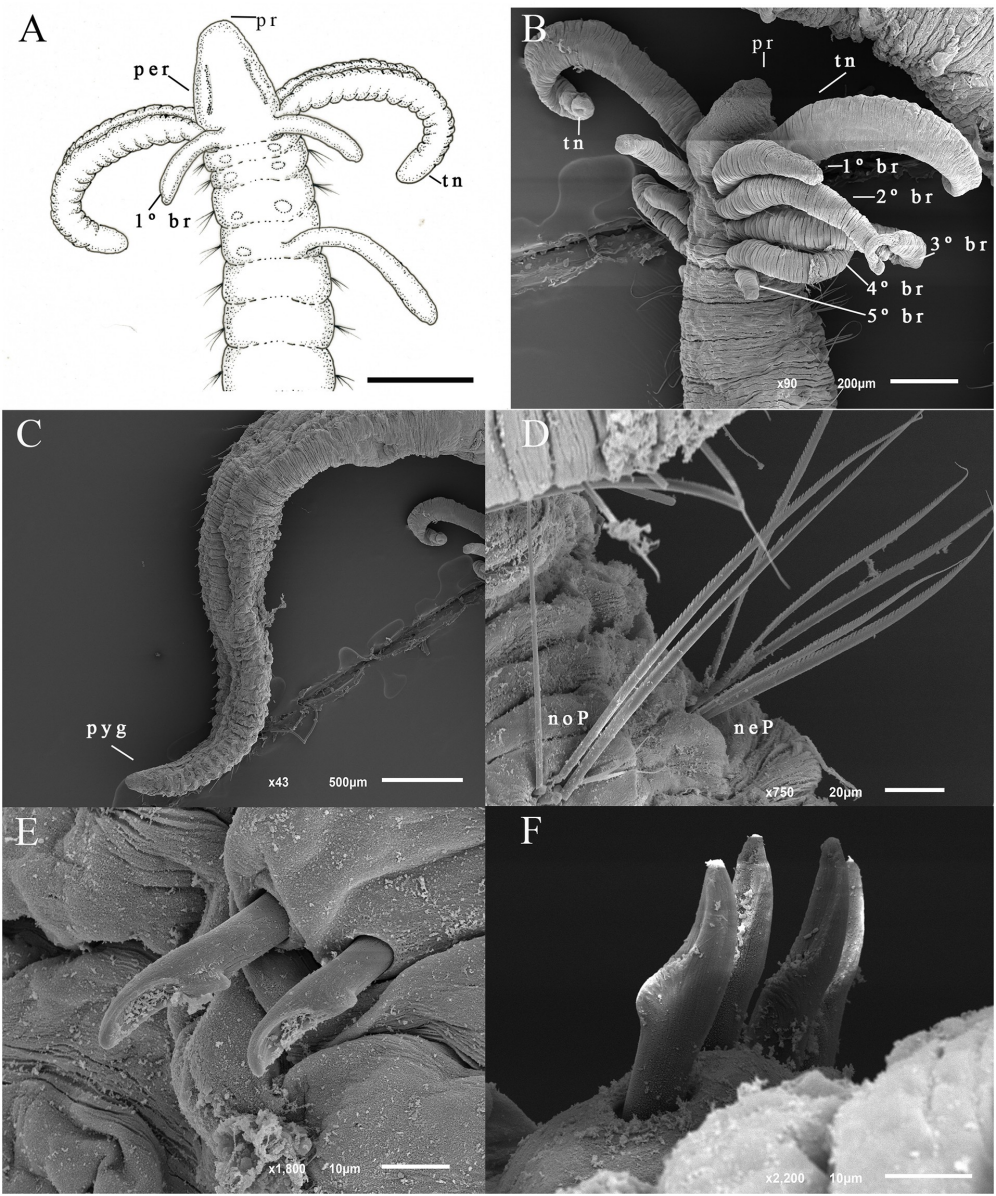
*Dodecaceria zelinhae* n. sp. (A) Anterior end, dorso-lateral view; (B) Anterior end, dorso-lateral view; (C) Posterior end, ventro-lateral view; (D) Notopodial and neuropodial serrated edge capillaries from chaetiger one; (E) Neuropodial hooks from posterior region; (F) Notopodial hooks from posterior region. Scale bars: 400 μm (B).

*Type locality*. Atlantic Ocean: Rocas Atoll (Brazil).

*Material examined*. BRAZIL: Rio Grande do Norte, Rocas Atoll, Farol Island; Holotype:–-3.860350°S, -33.815775°W, intertidal depths borrowed in coral, 10/05/15, (MNRJP-007567); Paratypes–-3.860350°S, -33.815775°W, intertidal depths, 09/19/15, two ind. prepared for SEM; 09/21/15, four ind., (MNRJP-007569); 10/05/15, one ind. prepared for SEM; one ind. (MNRJP-007568); Podes Crer pool, intertidal depths, 09/29/15, one ind. prepared for SEM.

*Description*. Largest specimen with 77 chaetigers, 18 mm long (from prostomium to pygidium). Anterior chaetigers wider than long (Fig 3A and 3B), posterior chaetigers short and flattened dorsoventrally (Fig 3C). Color in alcohol pale with brown pigments concentrated in posterior region. Prostomium rounded, peristomium longer (Fig 3A and 3B). Eyespots absent, nuchal organs not observed. Tentacles inserted laterally on peristomium. Five pairs of branchiae from peristomium to 4th chaetiger. The first pair of branchiae dorsal to tentacles in the peristomium, while t remaining branchial pairs are located from chaetiger 1 up to 4, dorsally to notochaetae (Fig 3A). Capillary chaetae with serrated edges present throughout body (Fig 3D). Neuropodium with 3–4 capillaries up to chaetiger 8, while subsequent chaetigers with one or no capillaries. Notopodium with 1–5 capillaries up to chaetiger 11, 1–3 from chaetiger 12. Neuropodial hooks from chaetiger 9 up to chaetiger 77, 1–3 hooks per fascicle. Neuropodial hooks short, wide, and more curved with deep apical concavity (Fig 3E). Notopodial hooks from the chaetiger 11 with 2–4 hooks per fascicle. Notopodial hooks long, slender with apical concavity shallow, somewhat spoon-shaped (Fig 3F). Pygidium conical (Fig 3C).

Variation in the number of pairs of branchiae: five (holotype), three (MNRJP-007568; MNRJP-007569), four (MNRJP- 007569).

#### Specimens in regeneration

**Fig 4:**
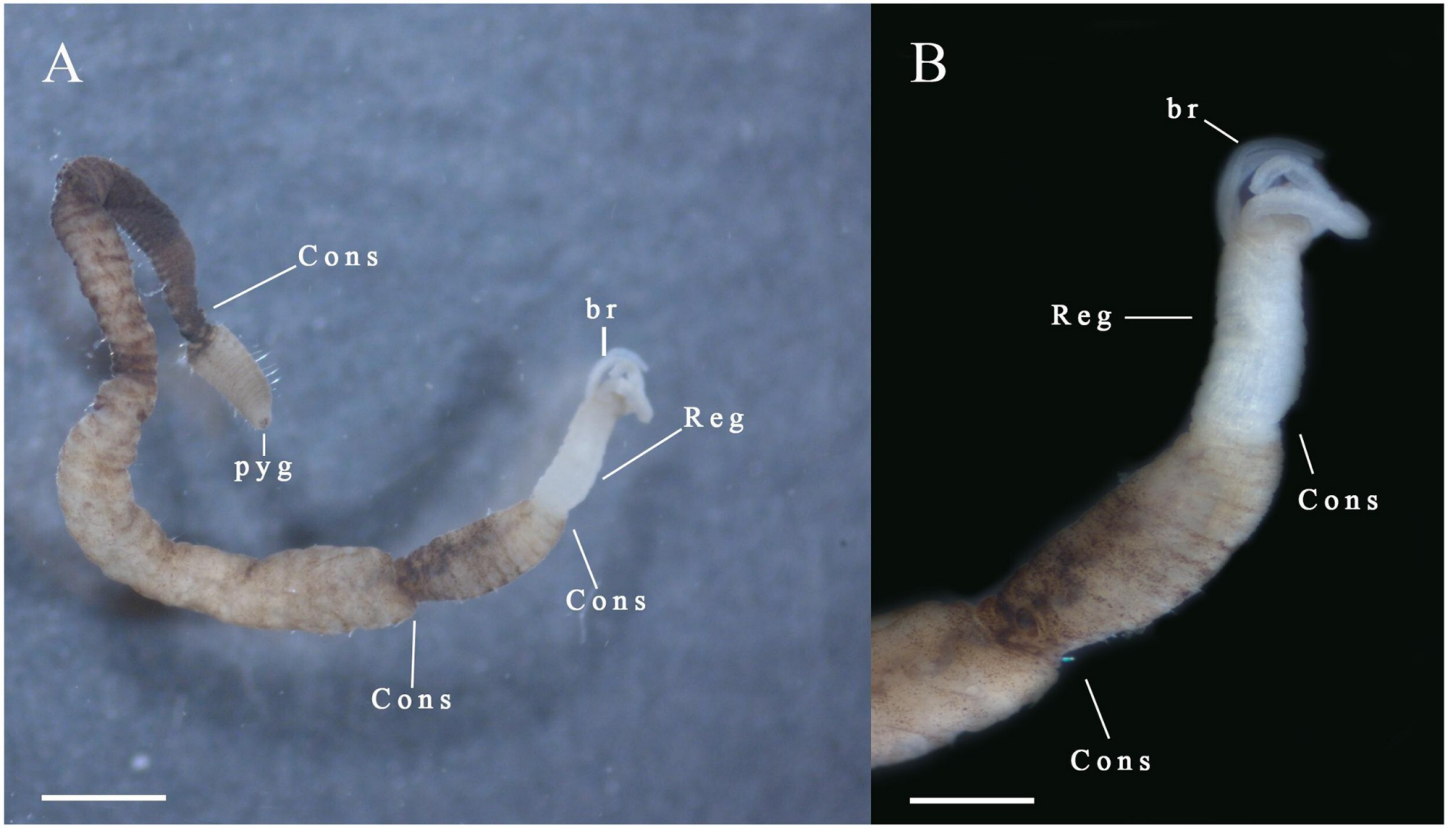
Regeneration in *D*. *zelinhae*. (A-B) *D*. *zelinhae* n. sp.; (A) Complete individual; (B) Anterior region, lateral view. Scale bars: (A) 1000 μm; (B) 500 μm.

One specimen with anterior regeneration (Fig 4A and 4B), and one specimen with both anterior and posterior regeneration (MNRJP-007569).

*Habitat*. Hard bottom and coral, intertidal depths.

*Distribution*. Presently known only from the Atlantic Ocean: Rocas Atoll (Brazil).

*Remarks*. *Dodecaceria zelinhae* n. sp. has a variable number of pairs of branchiae ranging from three to five. It is not unusual for *Dodecaceria* species to show variability in this character. For example, *D*. *concharum* Ørsted, 1843 from Denmark has 3–6 pairs, and *D*. *meridiana* Elías & Rivero, 2009 from the Argentinian coast has 7–18 pairs (Table 1). A few species have variation in the number of branchial pairs on the peristomium comparable to *D*. *zelinhae* n. sp. namely *D*. *alphahelixae* Blake & Dean, 2019 from the Caribbean Sea, Panama, with 2–4 pairs of branchiae; *D*. *saeria* Paterson & Neal 2020 from Malvinas islands, with 2–3 pairs of branchiae; and *D*. *pulchra* Day, 1955 from Atlantic Ocean, South Africa, with 4–5 pairs of branchiae. Although this variation in branchial pairs is comparable in this species, other characteristics set them apart. While *D*. *zelinhae* n. sp. the first hooks occur on chaetiger 9, whereas *D*. *alphahelixae* and *Dodecaceria saeria* have their hooks from the chaetiger 5 [9] and 22 [35], respectively. Additionally, *D*. *zelinhae* n. sp. and *D*. *alphahelixae* have different peristomium morphology; the first has a smooth peristomium whereas the second has one annular ring. There are also differences in terms of habitats. In contrast to *D*. *zelinhae* n. sp. which is from shallow waters, *D*. *saeria* has been collected from depths greater than 450 m [33]. Moreover, *D*. *zelinhae* n. sp. differs from *D*. *pulchra* on the presence of capillary chaetae. *D*. *pulchra* has capillary notochaetae and neurochaetae from chaetiger 1 to chaetigers 12–14; after chaetigers 12–14, only a variable number of subsequent chaetigers have capillary notochaetae, and capillary neurochaetae are absent [36]. On the other hand, *D*. *zelinhae* n. sp. has capillary notochaetae in all chaetigers. Thus, *D*. *zelinhae* n. sp. differs from these similar species due to this combination of characters: hooks from chaetiger 11 in the notopodia, hooks from chaetiger 9 in the neuropodia, a smooth peristomium, and notochaetae present in all chaetigers. Notably, evidence of anterior regeneration was found in a specimen of *D*. *zelinhae* n. sp. This species can be distinguished by its anterior narrowing towards the head, which is unpigmented, in contrast to the rest of the body.

**Table 1:** Taxonomic characters for species of *Dodecaceria*.

Species	Per ring	Position of the 1^st^ pair of br	Pairs of per br	Total of br	1^st^ noP with hooks	1^st^ neP with hooks	Pyg lobes	Habitat	Type locality
*D*. *alphahelixae* Blake & Dean, 2019	1	per	1	2–4	5th	5^th^	1	coral reef	Deer Island, Panama
*D*. *ater* (Quatrefages, 1866)	nob	per	1	4	Nob	Nob	nob	nob	Bréhat Island, France
*D*. *berkeleyi* Knox, 1972	1	per	1	3–4	10th	10^th^	nob	shells of *Cominella adspersa* (Bruguière, 1789)	Kaiteriteri Beach, New Zealand
*D*. *capensis* Day, 1961	1	per	2	4	9–13^th^	8–11^th^	nob	calcareous algae *Lithothamnion* Heydrich, 1897	False Bay, South Africa
*D*. *carolinae* Aguilar-Camacho & Salazar-Vallejo, 2011	1	per	1	12	14–19^th^	13–18^th^	1	limestone, corals *Porites* Link, 1807 and *Pavona Lamarck*, *1801*	Yucatán Peninsula, México
*D*. *choromytilicola* Carrasco, 1977	absent	per	2	13	11th	11^th^	7	shells of *Choromytilus chorus* (Molina, 1782)	Coliumo Bay, Chile
*D*. *concharum* Ørsted, 1843 (sensu Blake, 1996)	absent	1^st^ chaetiger	absent	3–6	9th	7^th^	2	shells	Albæk Bay, Denmark
*D*. *coralii* (Leidy, 1855)	nob	per	2	7	9th	9^th^	nob	coral *Astrangia poculata* (Ellis & Solander, 1786)	Narragansett Bay, USA
*D*. *dibranchiata* Blake & Dean, 2019	absent	per	1	2	9–12^th^	8–11^th^	1	coral reef and mangrove bank	Golfo de San Blas, Panama
*D*. *diceria* Hartman, 1951	absent	per	1	1–2	11th	11^th^	1	shell *Onustus* Swainson, 1840	Florida keys, USA
*D*. *fistulicola* Ehlers, 1901 (*sensu* Fauvel, 1953)	nob	nob	Nob	5–8	Nob	Nob	nob	coral reef	Pacific Ocean, Chile
*D*. *gallardoi* Carrasco, 1977	2	per	2	3	6th	6^th^	3	nob	Bay of Concepción, Chile
*D*. *inhamata* (Hoagland, 1919)	absent	per	1	5	Nob	Nob	nob	nob	Bermuda archipelago, UK
*D*. *joubini* Gravier, 1905	nob	per	1	6	8th	8^th^	nob	coral reef	Moucha Island, Djibouti
*D*. *laddi* Hartman, 1954	1	per	1	2	8th	8^th^	nob	coral reef	Marshall Islands
*D*. *meridiana* Elías & Rivero, 2009	absent	per	1	7–18	7–17^th^	7–15^th^	1	limestone with mussels	Mar del Plata, Argentina
*D*. *multifiligera* Hartmann-Schröder, 1962	2	1^st^ chaetiger	absent	22	6^th^	1th	nob	*Macrocystis* rhizoids	Punta Arenas, Chile
*D*. *opulens* Gravier, 1908	nob	per	1	7 or 14	8^th^	8^th^	nob	nob	Pacific Ocean, Peru
^1^*D*. *pacifica* (Fewkes, 1889)	absent	per	1	7–10	7th	7^th^	4	nob	California, USA
*D*. *pulchra* Day, 1955	absent	per	1	4–5	11–12^th^	9–10^th^	nob	calcareous algae *Lithothamnion* Heydrich, 1897	False Bay, South Africa
*D*. *saeria* Paterson & Neal, 2020	absent	per	1	2–3	22–29th	22–29^th^	1	soft bottoms	Malvinas Islands, UK
*D*. *saxicola* (Grube, 1855)	nob	per	1	3	15th	15^th^	nob	coral reef	Mediterranean Sea, Greece
*D*. *zelinhae* n. sp.	absent	per	1	3–5	11th	9^th^	1	hard bottom and coral	Rocas Atoll, Brazil

^1^Based on Moore’s descriptions (1909, 1923) and Berkeley & Berkeley (1954).

nob—Not observed.

*Etymology*. This species is named in honor of Maurizélia de Brito Silva (Zelinha), in recognition of her important work in protecting the Rocas Atoll. For over 30 years, she has been working for ICMBio as the head of the Rocas Atoll Biological Reserve.

## Discussion

The systematics of the genus *Dodecaceria* have been constantly revised. Gibson [27] concluded that the pattern of branchiae and chaetae correlates with the specimens’ size and proposed greater attention to the way of life and details of asexual reproduction for the genus. Differences in the stages of development or regeneration and reproductive strategies raised questions about synonyms or even misidentifications [37]. Asexual reproduction and regeneration change individuals’ morphology and, sometimes, even make it impossible to determine some characters, such as the number of branchiae, prostomium, and peristomial morphology. Aguillar-Camacho & Salazar-Vallejo [38] described *Dodecaceria carolinae*, proposing a diagnostic character for the genus, which is the presence of branchiae that are either monomorphic (i.e., of the same size) or dimorphic (i.e., of different sizes along the body). However, specimens collected at Rocas Atoll show different regenerative phases of the anterior region, which was reflected in the length of the branchiae. This observation is consistent with Gibson’s findings on the reproductive modes of *Dodecaceria* species deposited in Museums [27]. Therefore, we argue that this character can vary depending on the developmental stages of the individual, which differences in size and regenerative stage can illustrate. Current studies on the reproductive modes and ontogeny process of *Dodecaceria* species are needed to understand which branchiae related characters are most appropriate for identification at a specific level.

There are approximately 20 valid species of *Dodecaceria* [1]. However, only six species of *Dodecaceria* are recorded in South Atlantic waters. On the Argentine coast, the species *D*. *concharum* [39, 40], *D*. *multifiligera* Hartmann-Schröder, 1962 [41], *D*. *meridiana* Elías & Rivero, 2009 and *D*. *saeria* Paterson & Neal, 2020 are recorded. On the South African coast, the species *Dodecaceria capensis* Day, 1961 and *Dodecaceria pulchra* Day, 1955 are recorded. In Brazil, two species of *Dodecaceria*, namely *D*. *capensis* [15] and *D*. *concharum* [16], have been documented. The limited availability of taxonomic publications highlights the significance of having more studies that focus on the Brazilian coastline. Consequently, further investigations would be highly recommended to enhance our comprehension of these taxa. It is evident that there is a lack of knowledge regarding the diversity of the genus in the South Atlantic Ocean. The Brazilian and Argentine coasts, given the already known diversity of Cirratulidae, are considered potential areas with a high probability of harboring *Dodecaceria* species [31].

To date, the only *Dodecaceria* species described from oceanic islands is *D*. *laddi*, which was originally recorded in the Marshall Islands [34]. No *Dodecaceria* species have been described from Oceanic islands in the South Atlantic prior to this study. It is already known that these islands exhibit high levels of endemism in marine taxa [42–44], and are important breeding sites [45–48]. Despite the growing anthropic pressures [49], some of them still present coral reefs considered “pristine”, for example, Rocas Atoll [50, 51].

Before the present study, three Cirratulidae species had been documented in Rocas Atoll: *Timarete caribous* (Grube, 1859), *Timarete punctata* (Grube, 1859), and *Timarete ceciliae* Magalhães *et al*., 2014 [52]. This research expands our understanding of Cirratulidae in the Atlantic and Rocas Atoll by presenting a new record of *D*. *dibranchiata* Blake & Dean, 2019, and the description of a new species.

## References

[1] Blake J.A. & Magalhães W. 2019_7.3.1.5 Cirratulidae, Ryckholt, 1851. Pp. 339–397, In: Purschke G., Böggemann M. & Westheide W. (Eds.), Handbook of Zoology. Annelida. Volume 1: Annelida Basal groups and Pleistoannelida, Sedentaria I. i–xii, 1–480. De Gruyter, Berlin. (this URL only identifies the larger Pleistoannelida, not the individual chapters such as Cirratulidae)

[2] Read, G.; Fauchald, K. (Ed.) 2023. World Polychaeta Database. Cirratulidae Ryckholt, 1851 Accessed through: World Register of Marine Species at: https://www.marinespecies.org/aphia.php?p=taxdetails&id=919 on 2023-09-21

[3] Blake, J.A. 1996. Chapter 8. Family Cirratulidae. Pp. 263–384. In: Blake, J.A., Hilbig, B., & P.H. Scott (Editors). Taxonomic Atlas of the Santa Maria Basin and Western Santa Barbara Channel. Vol. 6. Annelida Part 3. Polychaeta: Orbiniidae to Cossuridae. Santa Barbara Museum of Natural History, *i-vii* + 480 pp.

[4] Winnepenninckx BMH, Yves VP, Thierry B. Metazoan relationships on the basis of 18S rRNA sequences: a few years later. American Zoologist. 1998; 38: 888–906.

[5] McHugh D. Molecular phylogeny of the Annelida. Canadian Journal of Zoology. 2000; 78: 1873–1884.

[6] Struck TH, Westheide W, Purschke G. Progenesis in Eunicida (“Polychaeta,” Annelida)—separate evolutionary events? Evidence from molecular data. Molecular Phylogenetics and Evolution. 2002; 25: 190–199. doi: 10.1016/s1055-7903(02)00231-2 12383760

[7] Ørsted AS, Anders S. Annulatorum danicorum conspectus. Hafniæ: Sumtibus Librariæ Wahlianæ; 1843. https://www.biodiversitylibrary.org/item/44441

[8] Blake JA. Bitentaculate Cirratulidae (Annelida, Polychaeta) collected chiefly during cruises of the R/V Anton Bruun, USNS Eltanin, USCG Glacier, R/V Hero, RVIB Nathaniel B. Palmer, and R/V Polarstern from the Southern Ocean, Antarctica, and off Western South America. Zootaxa. 2018; 4537 1: 1–130. 30647335 10.11646/zootaxa.4537.1.1

[9] Blake JA, Dean HK. New species of Cirratulidae (Annelida, Polychaeta) from the Caribbean Sea. Zootaxa. 2019; 4671 3: 301–38. doi: 10.11646/zootaxa.4671.3.1 31716040

[10] Elías R, Rivero MS, Orensanz JML. New species of *Monticellina* and *Chaetozone* (Polychaeta: Cirratulidae) in the SW Atlantic, and a review of *Monticellina* species. Journal of the Marine Biological Association of the United Kingdom. 2017; 97: 1553–1563.

[11] Saracho-Bottero MA, Elías R, Magalhães WF. Taxonomic revision of *Cirratulus* (Polychaeta: Cirratulidae) from the coasts of Argentina, with description of a new species. Journal of the Marine Biological Association of the United Kingdom. 2017; 97: 889–896.

[12] Elías R, Saracho-Bottero MA, Magalhães WF. Two new species of *Protocirrineris* (Polychaeta: Cirratulidae) from Brazil. Revista de Biología Tropical. 2019; 67: 81–91.

[13] Freitas R, Ribeiro RP, Ruta C. *Kirkegaardia* Blake, 2016 (Annelida: Cirratulidae) from Southeastern Brazil with description of nine new species. PLoS ONE. 2022; 17: 1–27. Available from: doi: 10.1371/journal.pone.0265336 35537464 PMC9090474

[14] Elías Rivero MS. Two new species of Cirratulidae (Annelida: Polychaeta) from Mar del Plata, Argentina (SW Atlantic). Zoosymposia. 2009; 2: 139–148.

[15] Costa DA, Fernandes HF, Silva FA, Christoffersen ML. Checklist de espécies de Polychaeta (Annelida) da Praia do Seixas, João Pessoa, Estado da Paraíba, Nordeste do Brasil. Revista Brasileira de Gestão Ambiental e Sustentabilidade. 2017; 48: 313–20.

[16] Amaral EHM. A endofauna de *Schizoporella unicornis* (Johnston, 1847) (Bryozoa), no litoral norte do Estado de São Paulo. M.Sc. Thesis, Universidade Estadual de Campinas. 1980. http://acervus.unicamp.br/index.asp?codigo_sophia=52618

[17] Kikuchi RKP. Atol das Rocas, Litoral do Nordeste do Brasil—Único atol do Atlântico Sul Equatorial Ocidental. In: Sítios Geológicos e Paleontológicos do Brasil. DNPM/CPRM; 2002. pp. 379–90.

[18] Decreto No 83.549, de 5 de Junho de 1979. Diário Oficial da República do Brasil Brasília, DF, Brasil; 1979 p. 8036.

[19] ICMBio. Plano de Manejo para a Reserva Biológica do Atol das Rocas. Ministério do Meio Ambiente; 2007.

[20] Paiva PC, Young PS. Echeverria CA. The Rocas Atoll, Brazil: a preliminary survey of the Crustacea and Polychaete fauna. Arquivos do Museu Nacional do Rio de Janeiro. 2007; 65(3): 241–250. Available from: https://www.labpoly.biologia.ufrj.br/PaivaYoungEcheverria2007.pdf

[21] Caullery M, Mesnil F. Les formes épitoques et l’évolution des Cirratuliens. In: Annales de l’Université de Lyon. Lyon: G. Masson; 1898. https://www.biodiversitylibrary.org/item/192923

[22] Dehorne A. La schizométamérie et les segments tétragemmes de “*Dodecaceria caulleryi*”, n. sp.. Bulletin Biologique de la France et de la Belgique. 1933; 67, 298–326.

[23] Petersen ME. Reproduction and development in Cirratulidae (Annelida: Polychaeta). Hydrobiologia. 1999; 402: 107–128.

[24] Zattara EE, Bely AE. Phylogenetic distribution of regeneration and asexual reproduction in Annelida: regeneration is ancestral and fission evolves in regenerative clades. Invertebrate Biology. 2016; 135: 400–414.

[25] Ribeiro RP, Bleidorn C, Aguado TM. Regeneration mechanisms in Syllidae (Annelida). Regeneration. 2018; 5: 26–42. doi: 10.1002/reg2.98 29721325 PMC5911452

[26] Knox GA. *Dodecaceria berkeleyi* n. sp., a Polychaete (Family Cirrutulidae) from New Zealand. Journal of the Fisheries Board of Canada. 1971; 2810: 1437–43.

[27] Gibson PH. Systematics of *Dodecaceria* (Annelida: Polychaeta) and its relation to the reproduction of its species. Zoological Journal of the Linnean Society. 1978; 633: 275–87.

[28] Gibson PH. Distribution of the Cirratulid Polychaetes *Dodecaceria fimbriata*, *D*. *concharum* and *D*. *diceria* in European Waters Between Latitudes 48°N and 70°N. Journal of the Marine Biological Association of the United Kingdom. 1996; 763: 625–35.

[29] Martin EA. Polymorphism and methods of asexual reproduction in the annelid, *Dodecaceria*, of Vineyard Sound. The Biological Bulletin. 1933; 651: 99–105.

[30] Gibson PH. The specific status of the two cirratulid polychaetes, *Dodecaceria fimbriata* and *D*. *caulleryi*, compared by their morphology and methods of reproduction. Canadian Journal of Zoology. 1979; 577: 1443–51.

[31] Elias R, Rivero MS. First new Dodecaceria (Polychaeta: Cirratulidae) species from the SW Atlantic (38ºS—57ºW, Argentina). Revista de Biologia Marina y Oceanografia. 2009; 441: 131–6.

[32] Gibson PH. Reproduction in the cirratulid polychaetes *Dodecaceria concharum* and *D*. *pulchra*. Journal of Zoology. 1977; 1821: 89–102.

[33] Hartman O. The littoral marine annelids of the Gulf of Mexico. Publications in Marine Science. 1951; 21: 391–7.

[34] Hartman O. Marine annelids from the northern Marshall Islands. Professional Papers of the US Geological Survey. 1954; 260: 615–44.

[35] Neal L, Paterson GLJ, Blockley D, Scott B, Sherlock E, Huque C, et al. Biodiversity data and new species descriptions of polychaetes from offshore waters of the Falkland Islands, an area undergoing hydrocarbon exploration. Zookeys. 2020; 938: 1–86. doi: 10.3897/zookeys.938.49349 32549744 PMC7286948

[36] Day JH. The Polychaeta of South Africa. Part 3. Sedentary Species from Cape Shores and Estuaries. Zoological Journal of the Linnean Society. 1955; 4228 7: 407–52.

[37] Gibson PH. Augmented descriptions and nomenclature of the cirratulid polychaetes *Dodecaceria fimbriata* and *D*. *concharum*. Journal of the Marine Biological Association of the United Kingdom. 2015; 954: 697–702.

[38] Aguilar-Camacho JM, Salazar-Vallejo SI. *Dodecaceria carolinae* n. sp. (Polychaeta: Cirratulidae), a shallow-water species from the northwestern Caribbean Sea. Scientia Marina. 2011; 751: 95–102.

[39] Elías R, Rivero MS, Vallarino EA. Sewage impact on the composition and distribution of Polychaeta associated to intertidal mussel beds of the Mar del Plata rocky shore, Argentina. Iheringia. Série Zoologia. 2003; 933: 309–18.

[40] Elías R, Bremec CS, Vallarino EA. Polychaetes from a southwestern shallow shelf Atlantic area (Argentina, 38 S) affected by sewage discharge. Revista Chilena de Historia Natural. 2001; 743: 523–31.

[41] Orensanz J. Los Anélidos poliquetos de la provincia biogeográfica magallánica. Catalogo de las especies citadas hasta 1974. Laboratorio de Comunidades Bentónicas-Gabinete abierto Sta. Clara del Mar Contribución Técnica. 1974; 1: 3–76.

[42] Paiva SV, Oliveira FRRD, Lotufo TMC. Ascidians from Rocas Atoll, northeast Brazil. Frontiers in Marine Science. 2015; 2: 39.

[43] Hachich NF, Bonsall MB, Arraut EM, Barneche DR, Lewinsohn TM, Floeter SR. Island biogeography: patterns of marine shallow-water organisms in the Atlantic Ocean. Journal of Biogeography. 2015; 4210: 1871–82. Available from: doi: 10.1111/jbi.12560

[44] Barroso CX, Lotufo TMC, Bezerra LEA, Matthews-Cascon H. A biogeographic approach to the insular marine ‘prosobranch’ gastropods from the southwestern Atlantic Ocean. Journal of Molluscan Studies. 2016; 824: 558–63. Available from: doi: 10.1093/mollus/eyw015

[45] Wetherbee BM, Gruber SH, Rosa RS. Movement patterns of juvenile lemon sharks *Negaprion brevirostris* within Atol das Rocas, Brazil: A nursery characterized by tidal extremes. Marine Ecology Progress Series. 2007; 343: 283–93.

[46] Serafini TZ, França GB, Andriguetto-Filho MJ. Brazilian oceanic islands: known biodiversity and its relation to the history of human occupation. Journal of integrated Coastal Zone Management. 2010; 310: 281–301.

[47] Bellini C, Santos AJB, Grossman A, Marcovaldi MA, Barata PCR. Green turtle (Chelonia mydas) nesting on Atol das Rocas, north-eastern Brazil, 1990–2008. Journal of the Marine Biological Association of the United Kingdom. 2013; 934: 1117–1132.

[48] Bouth HF, Leite TS, Lima FD, Oliveira JEL. Atol das Rocas: An oasis for *Octopus insularis* juveniles (Cephalopoda: Octopodidae). Zoologia. 2011; 281: 45–52.

[49] Halpern BS, Frazier M, Potapenko J, Casey KS, Koenig K, Longo C, et al. Spatial and temporal changes in cumulative human impacts on the world’s ocean. Nature Communications. 2015; 61: 7615. Available from: doi: 10.1038/ncomms8615 26172980 PMC4510691

[50] Longo GO, Morais RA, Martins CDL, Mendes TC, Aued AW, Cândido D V, et al. Between-Habitat Variation of Benthic Cover, Reef Fish Assemblage and Feeding Pressure on the Benthos at the Only Atoll in South Atlantic: Rocas Atoll, NE Brazil. PLoS ONE. 2015; 106: e0127176. Available from: doi: 10.1371/journal.pone.0127176 26061735 PMC4464550

[51] Leão ZMAN, Kikuchi RKP, Ferreira BP, Neves EG, Sovierzoski HH, Oliveira MDM, et al. Brazilian coral reefs in a period of global change: A synthesis. Vol. 64, Brazilian Journal of Oceanography. 2016; 64: 97–116.

[52] Magalhães WF, Seixas VC, Paiva PC, Elias R. The Multitentaculate Cirratulidae of the Genera *Cirriformia* and *Timarete* (Annelida: Polychaeta) from Shallow Waters of Brazil. PLoS ONE. 2014; 911: e112727. Available from: doi: 10.1371/journal.pone.0112727 25393759 PMC4231064

