# Correction: New species and record of *Dodecaceria* (Annelida: Cirratulidae) the Biological Reserve of Rocas Atoll, Brazil, the only atoll in the South Atlantic Ocean

**DOI:** 10.1371/journal.pone.0343045

**Published:** 2026-02-13

**Authors:** Christine Ruta, Davi Moreira Mundim, Roberta Freitas, Rannyele P. Ribeiro

The word “from” is missing from the title. The correct title is: New species and record of *Dodecaceria* (Annelida: Cirratulidae) from the Biological Reserve of Rocas Atoll, Brazil, the only atoll in the South Atlantic Ocean. The correct citation is: Ruta C, Mundim DM, Freitas R, Ribeiro RP (2023) New species and record of *Dodecaceria* (Annelida: Cirratulidae) from the Biological Reserve of Rocas Atoll, Brazil, the only atoll in the South Atlantic Ocean. PLoS ONE 18(10): e0293087. https://doi.org/10.1371/journal.pone.0293087.
